# The Impact of Medical Waste on Indigenous Communities in Balochistan Pakistan: Sustainable Solutions in Reducing Inequality and Improving Resilience in Communities

**DOI:** 10.5334/aogh.4609

**Published:** 2025-04-25

**Authors:** Shakir Ullah, Usman Khan, Qasim Jan, Taher Saifuddin

**Affiliations:** 1 Faculty of International Studies, Henan Normal University, Xinxiang, Henan, P.R, China; 2 Department of Development Studies, National University of Science and Technology (NUST), Islamabad, Pakistan; 3 School of Education, Zhejiang Normal University, Zhejiang, China; 4 MacMillian Institute of Applied Health Sciences 55 Town Center Court Suit 700 Scarborough Toronto, Canada

**Keywords:** Medical Waste Management, Inequality, Indigenous communities, Pakistan

## Abstract

*Aim:* The study investigated the adverse impact of medical waste on Indigenous communities and explored sustainable solutions to reduce inequality and enhance resilience.

*Methods:* We adopted a qualitative thematic analysis from primary data collected from 176 respondents. Data were collected through focus groups and interviews.

*Results:* In the research, we examined the interconnected themes challenging medical waste management practices in Pakistan, such as poor disposal practices, health and environmental hazards of poor medical waste management through disease transmission and infections, and soil and water contamination. Socioeconomic disparities and inequality were also identified, resulting in economic burdens on vulnerable groups—the theme of which was Indigenous communities disadvantaged through health risks and vulnerabilities and disproportionate impact on health and well‑being. Themes further highlight government efforts, suggested regulatory and policy reforms, capacity building, and awareness.

*Recommendations:* We developed three significant recommendations for sustainable solutions to reducing inequality and improving community resilience. The first is community empowerment and awareness, emphasizing the need to educate community members, healthcare professionals, and waste handlers about the risks of improper medical waste disposal. The second is strengthening infrastructure and collaboration, highlighting the urgent need to establish proper waste collection and segregation infrastructure. Collaboration among healthcare facilities, waste management agencies, government bodies, and community leaders is instrumental in designing comprehensive solutions that meet the unique needs of Indigenous communities. The third one is policy enhancement and enforcement, suggesting the importance of policy revisions and rigorous enforcement mechanisms. The study advocates for policies that reflect current challenges and encourage innovative approaches to medical waste management.

## 1. Introduction

The increased medical technology and healthcare facilities worldwide in recent years have undoubtedly improved life expectancy and human well‑being. However, the expanding healthcare industry also poses considerable challenges, especially with regard to the disposal of medical waste. Medical waste management needs urgent attention from academics, decision‑makers, and communities worldwide because it poses serious risks to both human health and the environment. Medical waste refers to materials generated by healthcare facilities, such as hospitals, clinics, and laboratories, that can be potentially hazardous to human health and the environment. This waste includes sharps (needles, syringes), expired medications, used personal protective equipment (PPE), and various chemical and pharmaceutical products. Considering a developing nation such as Pakistan, a country with a diversified cultural fabric and a rich Indigenous community tradition, the issue of medical waste becomes intriguing owing to its complex social and environmental implications. Indigenous groups in Pakistan have always protected their ecosystems and cultural practices as stewards of the environment [1]. These communities bear a disproportionate weight from the effects of medical waste despite having an innate connection to nature. Their sensitivity to the negative impacts of improperly handled medical waste is made worse by their marginalized status, combined with their limited access to resources and services [2]. Some of the challenges faced by people owing to this issue include health risks, where disposing of medical waste improperly can result in the spread of infectious diseases and endanger the health of Indigenous populations [3]. Environmental contamination, where inappropriate disposal of healthcare waste can harm nearby ecosystems, including water supplies and farmland, is also risky. The livelihoods of Indigenous groups, which frequently rely on natural resources for sustenance, may be impacted by this contamination [4].

The handling of medical waste is a very important topic in the healthcare sector; similar to many other nations, Pakistan has seen a considerable rise in the use of personal protective equipment (PPE) owing to the coronavirus disease 2019 (COVID‑19) epidemic. An astounding 141 million units of PPEs have reportedly been distributed across hospitals and government agencies throughout Pakistan, according to statistics given by the National Disaster Management Authority of Pakistan (NDMA). These PPEs were obtained via direct purchases and international contributions, and they included different kinds of facemasks, protective suits, goggles, gloves, surgical caps, face shields, and shoe coverings. During the pandemic, over 5000 tons of PPE waste were produced in government hospitals due to the widespread usage of PPEs [5]. Studies have highlighted the need for better methods and infrastructure by shedding light on the unique issues related to medical waste management in Karachi, Lahore, Peshawar, and Balochistan. Hospital waste management, disposal, and segregation procedures in Karachi are major issues [6]. The environment and public health may be at risk when hazardous and non‑hazardous waste is mixed due to improper waste segregation. Developing efficient waste segregation systems is essential to guarantee the secure management and disposal of various waste streams. Tertiary hospitals in Karachi have been shown to have poor waste management methods [7].

The difficulties in handling medical waste resulting from inadequate storage facilities and poor transportation practices underscore the need for better waste management systems and procedures in healthcare institutions. Peshawar’s waste management procedures have been scrutinized by research that suggests that teaching hospitals have insufficient waste management procedures, and the lack of incinerators in densely populated locations makes it difficult to dispose of medical waste safely and effectively [8]. A recent systematic review by Fadaei [9] highlighted the need to adopt proper waste management techniques despite the need for more research on medical waste management in Balochistan. This research examines medical waste’s various and complex effects on Indigenous populations in Balochistan, Pakistan. This study aims to suggest sustainable solutions that reduce inequality and enhance resilience by recognizing the precise difficulties these communities encounter. This project aims to contribute to developing practical and culturally appropriate interventions for Indigenous populations in Balochistan using the districts of Gwadar, Kech, and Quetta as case studies.

### 1.1 Effects of poor waste management on local indigenous communities

Improper handling of medical waste severely affects local Indigenous people in Pakistan, harming the environment, contributing to socioeconomic factors, and impacting public health. From a public health perspective, improper disposal can spread infectious diseases among Indigenous communities. Environmentally, mismanaged medical waste can contaminate groundwater and soil, disturbing the natural ecosystem and affecting local flora and fauna [10, 11]. Restricted access to healthcare services exacerbates the issue for Indigenous populations. In regions with inadequate healthcare facilities, people exposed to hazardous waste may not receive timely treatment, leading to increased health issues and avoidable fatalities [12]. Indigenous groups such as the Baloch and Pashtun are particularly vulnerable owing to their cultural and traditional practices. The Baloch, who lead a nomadic life in isolated regions, have limited access to healthcare and waste management facilities, making them more susceptible to the risks associated with improper waste disposal [13, 14]. Similarly, the Pashtun community in Balochistan, reliant on natural resources for survival, is directly affected by contamination from inadequate waste disposal, which threatens their water supplies and agricultural lands [15, 16].

### 1.2 Indigenous communities – most affected

Indigenous peoples are culturally distinct groups with ancestral ties to their lands [17]. Balochistan, Pakistan’s largest province, is home to Indigenous communities such as the Baloch and Brahui, who maintain centuries‑old cultural, linguistic, and territorial traditions [18]. Despite their strong ties to ancestral lands and environment, they face systemic marginalization, including poor access to healthcare, education, and infrastructure. Nonetheless, they preserve rich oral histories and cultural practices vital to the region’s identity [19, 20]. Often residing in remote, underserved regions, they face limited healthcare access, increasing their vulnerability to the adverse effects of poor medical waste management [21, 22]. The closeness of waste disposal sites to Indigenous villages in remote areas leads to careless dumping and poor segregation, contaminating soil and water. This environmental pollution poses serious health risks to these communities [22]. Inadequate waste management facilities exacerbate the issue, as these areas may lack regular waste collection or hazardous material treatment services. The accumulation of waste near inhabited areas increases exposure to toxic substances and hazardous viruses, posing serious public health risks [22].

### 1.3 Government measures to manage medical waste

The Pakistani government has implemented various strategies to manage medical waste and mitigate its adverse effects on public health and the environment. These strategies include institutional, operational, and regulatory activities to improve waste management standards. A key initiative is the creation of comprehensive waste management legislation and policies. The government established the National Disaster Management Authority (NDMA) to coordinate and oversee disaster management, including medical waste handling during pandemics and other crises [5]. The NDMA collaborates with the Ministry of Health, provincial health departments, and waste management authorities to develop and implement medical waste management plans during emergencies [23, 24]. The government also runs training programs for healthcare workers and waste management professionals to enhance compliance with waste management standards. These programs cover proper waste handling, the use of personal protective equipment (PPE), waste segregation [8], and infectious waste management [25]. Hassan et al. [26] state that these initiatives aim to improve waste‑handling procedures’ overall effectiveness and safety. Public awareness campaigns, such as the Clean Green Campaign, emphasize responsible waste disposal and its impact on health and the environment [27]. The government also banned single‑use plastic bags, with enforcement supported by educational campaigns and alternative bag distribution [28]. These efforts encourage behavioural changes and community involvement in waste management [29].

### 1.4 Challenges to managing medical waste

In Pakistan, several obstacles hinder effective medical waste management. One major issue is the lack of regulatory enforcement and compliance. Although the government has established waste management regulations, their implementation is inconsistent across regions. Gill et al. [5] note that poor monitoring and control in healthcare facilities lead to non‑compliance with waste handling protocols, allowing improper disposal practices that harm human health and the environment. Insufficient waste management infrastructure further exacerbates the problem. Many healthcare institutions, especially in rural areas, lack access to necessary waste treatment facilities and equipment, such as incinerators and autoclaves, making safe hazardous waste disposal difficult [30]. Additionally, inadequate waste collection services contribute to environmental contamination and health risks. Torkashvand et al. [3] highlight that limited financial resources in healthcare often prioritize other urgent needs over waste management, leaving budgets insufficient for proper waste segregation, transportation, and treatment. The high costs of establishing waste treatment facilities and advanced technologies strain already scarce resources.

## 2. Methodology

### 2.1 Research design

This research was conducted to investigate the adverse impacts of medical waste on Indigenous communities in Balochistan, Pakistan, and to propose sustainable solutions to reduce inequality, improve resilience, and address the health, environmental, and socioeconomic challenges caused by improper medical waste management. This study employs a qualitative research design, specifically a thematic analysis research approach, to explore the impact of medical waste on Indigenous communities in Pakistan. A qualitative approach was selected to explore the lived experiences of Indigenous communities in Balochistan, providing in‑depth understanding of the health, environmental, and socioeconomic impacts of medical waste and developing context‑specific, culturally sensitive, and sustainable solutions to address these challenges. We adopted purposive sampling to select participants from three Indigenous districts in Balochistan, Pakistan, namely Gwadar, Kech, and Quetta. The selection criteria for participants focused on individuals who have direct experiences or knowledge related to medical waste issues. The respondents comprised medical staff, administrative and laboratory staff, pharmaceutical companies’ staff, local Indigenous community leaders, healthcare workers, waste management personnel, and environmental experts. The research data were collected through semi‑structured interviews and eight focus group discussions featuring 12 respondents each. The number for each focus group discussion has been validated by previous research [31, 32]. Three focus groups were conducted in the Kech and Quetta districts, while two focus group discussions were held in the Gwadar district. The key consideration in deciding the number of focus groups in each district was the population size of the districts. According to the Pakistan Bureau of Statistics [33], Quetta (2,595,492) and Kech (1,060,931) both have populations exceeding one million, while Gwadar (305,160) has a far smaller population – hence the decision by the researchers to use two focus groups, unlike the other two districts that had three each. Thus, 96 respondents participated in the focus group discussions.

In all, 80 semi‑structured interviews were conducted to elicit responses from the interviewees in the three selected districts. Using the population again as our criteria, 30 interviews were conducted in Kech and Quetta each, while 20 were conducted in Gwadar. Scholars have opined that at least 20 to 40 interviews were required to uncover cross‑cultural meta‑themes across different locations [34, 35]. The researchers relied on these authorities in designing the data collection procedure. The focus group talks and interviews were recorded to enhance the validity and reliability of the data collection process. The researchers utilized notebooks to record observations during the data collection procedures. Audio and video recordings using mobile devices were used, ensuring that important details were documented *in situ*, thereby reducing the risk of omission. The researchers determined that the number of interviews and focus group discussions conducted was sufficient to reach data saturation. Therefore, no additional participants were included, as no new themes or insights were emerging. The major challenges faced by the researchers in the data collection process were conflicting schedules for participants and interviewees. Some interviewees had last‑minute emergencies leading to rescheduling of the interviews; 14 such incidents were recorded. In the focus group discussion sessions, some participants were forced to exit discussion sessions owing to work‑related emergencies and rejoined after some minutes, with the highest staying away for 20 minutes before rejoining. Three such incidents were recorded. The focus group sessions lasted between 2 hours and 2 hours 25 minutes, while the interviews lasted between 1 and 1.5 hours. The field data collection period spanned eight months, commencing on January 1, 2023, and concluding on August 31, 2023.

### 2.2 Data analysis

The thematic analysis process was guided by Braun and Clarke’s six‑step approach to ensure a systematic and rigorous analysis. The first step was familiarization with data, which involved decoding the interviews to gain a comprehensive understanding. The next step was initial coding, developed by identifying recurring patterns, themes, and concepts in the data. The third step involved searching for themes, which involved identifying potential themes related to the impact of medical waste on Indigenous communities and the sustainable solutions proposed by participants. Reviewing themes was then conducted, where the identified themes were reviewed and refined. Themes were constantly compared and contrasted to ensure consistency and coherence. Then, defining and naming themes was conducted, where final themes were clearly defined, and representative quotes from the data were chosen to illustrate each theme effectively. The last report was written in an organized and coherent manner, providing a clear and detailed account of the impact of medical waste on Indigenous communities in Pakistan and the sustainable solutions identified.

### 2.3 Ethical considerations

The Institutional Review Board (IRB) of Henan Normal University reviewed and approved this research with approval number HN‑FIS‑073. The study complies with ethical standards as outlined by university policies and international research ethics guidelines. First, informed consent was received from all the participants prior to data collection, with the study’s purpose, procedures, and the confidentiality of their responses. Written consent was obtained from each participant. Second came anonymity and confidentiality, where the participants’ identities and potentially identifying information were kept strictly confidential throughout the study. Pseudonyms were used in the reporting of findings to ensure anonymity.

## 3. Results

The study conducted a thematic analysis of the data gathered through interviews with the respondents. The themes identified are discussed with their sub‑themes on each major subheading on which the data were collected. Table 1 summarizes the respondents’ demographic information, combining the 96 focus group participants and 80 interviewees.

**Table 1:** Demographic distribution of the study participants.

VARIABLES		FREQUENCY	PERCENTAGE (%)
Gender	Female	62	35
	Male	114	65
Age (years)	20–30	22	13
	30–40	90	51
	40+	64	36
Education	High school and below	8	4
	University education	137	78
	Postgraduate	31	18
Profession	Medical staff (doctors and nurses)	18	10
	Hospital administrative staff	11	6
	Laboratory staff	21	12
	Pharmaceutical companies’ staff	15	9
	Local Indigenous community leaders	37	21
	Caregivers	18	10
	Waste management personnel	32	18
	Environmental experts	24	14
	**Total**	**176**	**100**

*Source*: Authors compilations from field data.

Table 1 presents the demographic characteristics of the sample population. The majority of the participants were male (65%), which may influence the findings by reflecting a predominantly male perspective. The age distribution revealed that 51% of the respondents were between 30 and 40 years old, whereas the 20–30 age group was the smallest at 13%. The sample was generally well‑educated, with 78% holding university degrees, suggesting a strong capacity for understanding waste management issues. Local Indigenous community leaders were the most significant group (21%), followed by waste management personnel (18%) and laboratory staff (12%). Medical staff and caregivers each represented 10% of participants, contributing a moderate proportion relative to other occupational categories. Codes were used to identify the respondents during analysis; the legend for the codes is provided in Table 2.

**Table 2:** Code for identifying respondents.

VARIABLES		CODE
Gender	Female	F
	Male	M
Age (years)	20–30	1
	30–40	2
	40+	3
Education	High school and below	H
	University education	U
	Postgraduate	P
Profession	Medical staff (doctors and nurses)	M
	Hospital administrative staff	H
	Laboratory staff	L
	Pharmaceutical companies’ staff	P
	Local Indigenous community leaders	I
	Caregivers	C
	Waste management personnel	W
	Environmental dxperts	E

*Source*: Authors compilations from field data.

For example, a female respondent in the age range of 30–40 years with university education and hospital administrative staff was coded as F2UH.

### 3.1 Problem of medical waste management in pakistan districts

The study first investigated the problem of medical waste management in Pakistan, with a keen focus on three districts in the Balochistan region. The various themes that emerged are outlined below.

### 3.2 Theme 1: A challenge in medical waste management

The first theme identified from the results is that there is a challenge in medical waste management in Pakistan. Two major subthemes supported this.

#### 3.2.1 Subtheme: Poor disposal practices

This implies that the disposal of medical waste is not appropriate. We found no designated collection points for medical waste in many areas. As suggested by **Respondent 23 (M2PM)**, “…hospitals often resort to burning waste or mixing it with regular trash due to the absence of proper facilities.” **Respondent 14 (M3UE)** also indicated, “The current situation is worrisome as medical waste often ends up in open dumps or rivers, causing environmental degradation.”

#### 3.2.2 Subtheme: Poor waste management

Another aspect that was evident was the poor medical waste management by all the stakeholders involved, including the health facilities and the Pakistan district’s waste management systems. For instance, **Respondent 67 (F1UW)** indicated, “Healthcare workers receive minimal training on waste segregation, and this impacts the entire waste management process.” There needs to be a comprehensive arrangement from the facilities to the city management on how the waste should be handled, an aspect that is lacking. **Respondent 5 (F2UW)** indicated that “We need advanced equipment for safe disposal, but many healthcare institutions can’t afford such investments….”

### 3.3 Effects of medical waste management

The research investigated the effects of poor medical waste management on local Indigenous communities in Pakistan and extended the analysis to evaluate the effects on health and socioeconomic inequalities. The themes developed are discussed below.

#### 3.3.1 Theme 2: Health and environmental hazards

The major effects identified were health and environmental hazards and adverse effects from poorly disposed medical waste. This was derived from two subthemes.

#### 3.3.2 Subtheme: Disease transmission and infections

Diseases and infections were reported to have spread among the local communities owing to poor waste disposal. As indicated by **Respondent 89 (F1PM)**, a community elder, “We have seen an increase in cases of skin infections and gastrointestinal illnesses due to the open dumping of medical waste near our village.” It was also found that “Improper disposal of used syringes and contaminated materials has led to concerns about the spread of diseases like hepatitis and HIV.” (**Respondent 73, M1UM**).

#### 3.3.3 Subtheme: Soil and water contamination

Medical waste ends up on open ground and in water bodies, contaminating them. According to a **health expert, Respondent 64 (M3PM),** “Heavy metals and chemicals from medical waste have seeped into the soil, affecting crop quality and posing long‑term health risks.”

### 3.4 Theme 3: Socioeconomic disparities and inequality

This theme emerged from the disproportionate burden faced by vulnerable groups and their limited access to healthcare services. The responses illuminate how children, the elderly, and waste pickers are disproportionately affected, highlighting the social injustices associated with inadequate waste management.

#### 3.4.1 Subtheme: Vulnerable groups

Children and the elderly were considered the most affected by medical waste that had been improperly disposed of. As stated by a health worker, **Respondent 82 (F2UH)**, “Children and the elderly are especially vulnerable to medical waste‑related illnesses, as they often come into direct contact with waste materials.” Additionally, **Respondent 49 (M3HI)** suggested that “… the waste pickers, mostly from our community, are risking their health by scavenging through the medical waste for a meager income.”

#### 3.4.2 Subtheme: Economic burden

The affected communities bear additional financial burdens to address the negative impacts of poor medical waste management and disposal, such as disease outbreaks. For instance, **Interviewee 72 (M3UI)** indicated, “… Our community’s resources are already limited. Spending money on treating illnesses caused by medical waste leaves us with less for education and other basic needs.” Additionally, the people living in rural areas consider themselves marginalized, as indicated by **Respondent 38 (M1UI)**: “…The lack of waste management facilities deepens the divide between us and urban areas, perpetuating our marginalization.”

### 3.5 Theme 4: Disadvantaged indigenous communities

This theme introduces the concept that Indigenous communities are among the most affected by medical waste that is improperly disposed of. Two subthemes emerged: (1) health risks and vulnerabilities and (2) cultural practices and their health impact.

#### 3.5.1 Subtheme: Health risk and vulnerabilities

This theme implies that Indigenous communities suffer owing to inadequate healthcare access, cultural practices and contamination of vital resources. **Interviewee 62 (M3HI)** stated that “Indigenous communities often lack proper healthcare access. Improper medical waste disposal increases the risk of infectious diseases, disproportionately affecting their health.”

#### 3.5.2 Subtheme: Disproportionate impact on health and well‑being

Indigenous communities experience greater health challenges compared with other populations, primarily owing to systemic inequalities and environmental exposure. For instance, **Interviewee 53 (F3PM)** indicated that “….indigenous communities, often residing in remote areas, lack proper waste management infrastructure. This exposes them to higher health risks due to the proximity of medical waste to living spaces.” Additionally, “…Limited healthcare access in these communities magnifies the impact of improper waste disposal, leading to increased susceptibility to diseases.” (**Respondent 96, F2UM**).

### 3.6 Government measures to manage medical waste

This section evaluates the efforts and measures implemented to manage medical waste in Balochistan’s districts. The analysis is organized into two categories: government‑led initiatives and efforts undertaken by local and non‑governmental organizations (NGOs).

#### 3.6.1 Theme 5: Regulatory and policy reforms

The first theme identified was the regulatory and policy reforms implemented by the government. This theme is primarily explained through two subthemes: (1) policy integration and alignment and (2) strengthening and enforcement of regulations

#### 3.6.2 Subtheme: Policy integration and alignment

This involves the policies that have been adopted and implemented by the government to address the issue of medical waste management. For instance, **Respondent 17 (M3PE)** indicated that there was a need for “… regulations outlining clear responsibilities for healthcare facilities, waste generators, and waste management agencies.” Another health worker, **Respondent 89 (M3PE)**, indicated that “…… the Pakistan government has established the National Disaster Management Authority (NDMA) as a body to address such issues. NDMA should further integrate medical waste management into urban planning can ensure proper waste segregation and disposal.”

#### 3.6.3 Subtheme: Strengthening and enforcement regulations

This subtheme reflects the perceived need for more robust legal frameworks and enforcement mechanisms to address the challenges of medical waste management. **Respondent 62 (F2UE)** pointed out that, “while policies exist, enforcement remains inconsistent, especially in remote and underserved areas.” Similarly, **Respondent 95 (M3UE)** stressed the importance of regulatory oversight: “…. Monitoring and penalties must be increased. If hospitals or clinics are not disposing of waste properly, there should be consequences.”

### 3.7 Theme 6: Capacity building and awareness

This theme emerged from the effort aimed at raising awareness among stakeholders regarding the importance of proper medical waste management. It is supported by two subthemes: (1) public awareness campaigns and (2) resource allocation and collaborations.

#### 3.7.1 Subtheme: Public awareness campaigns

Public awareness regarding the need for effective medical waste management is primarily driven by local organizations and NGOs. As suggested by **Respondent 48 (M1UE)** “…..most healthcare NGOs are ‘conducting educational campaigns to inform the public about the dangers of improper medical waste disposal and the importance of segregation.’” In addition, there is also “… training for the healthcare professionals on comprehensive training on proper waste handling to minimize risks.” (**Respondent 59, F1UE**).

#### 3.7.2 Subtheme: Collaboration and resource allocation

Both government and the local organizations are making efforts to collaborate and pool resources to address this issue. According to **Respondent 72 (M3UI)**, “….the allocation of budgetary resources to waste management infrastructure is playing a role in improved waste collection, segregation, and disposal.” Additionally, “there is increasing effort by the government agencies, healthcare institutions, waste management companies, and NGOs to collaborate for a holistic solution.”

### 3.8 Recommendations of sustainable solutions, measures and strategies

Following an investigation into the prevailing conditions of medical waste management in Balochistan’s districts of Gwadar, Kech, and Quetta and its impact on Indigenous communities, the study further explored the sustainable solutions, measures, and strategies to the issue. The themes presented below were developed on the basis of the responses of the interview participants.

#### 3.8.1 Theme 7: Community empowerment and awareness

The first recommendation theme focused on raising awareness among Indigenous communities regarding medical waste management. The following sub‑themes supported this theme.

#### 3.8.2 Subtheme: Education and training

This subtheme highlights the importance of emphasizing the education of community individuals, medical personnel, and waste handlers regarding proper handling, segregation, and disposal of medical waste. For instance, **Respondent 65 (F2UH)** indicated, “…..healthcare staff should receive training on safe disposal practices. This would empower them to take the lead in ensuring medical waste is managed responsibly.”

#### 3.8.3 Subtheme: Cultural sensitivity

This aspect implied the need for designing solutions that align with the cultural values, beliefs, and practices of local communities. Such interventions require acknowledging and respecting societal norms. According to **Respondent 81 (M3HI)** “…..It’s crucial to approach the issue in a way that doesn’t clash with our cultural practices. Our elders appreciated when waste management was presented in a culturally sensitive manner.”

### 3.9 Theme 8: Strengthening of collaborations and structures

The second recommendation emphasizes the need to establish effective collaborations and strengthen the existing structure through multi‑stakeholder engagement and infrastructure improvement.

#### 3.9.1 Subtheme: Multi‑stakeholder collaboration

Multi‑stakeholder collaboration implies emphasizing coordinated collaboration among healthcare facilities, waste management companies, governmental organizations, and community leaders to handle medical waste management comprehensively. Many interviewees highlighted this need; for instance, one participant noted, “…if government officials, healthcare providers, and community leaders came together to form a committee focused on improving waste management, this collective effort would make a significant impact.” **(Respondent 89, M3PE)**.

#### 3.9.2 Subtheme: Improving infrastructure

This subtheme highlights the urgent need for the government to address the inadequate waste collection and disposal infrastructure in Indigenous communities. **Respondent 67 (M3UW)** emphasised that “…health facilities should collaborate with local authorities to set up designated collection points for medical waste. This would reduce the chances of waste being discarded haphazardly.”

### 3.10 Theme 9: Policy enhancement and enforcement

The third recommendation focuses on enhancing and enforcing policies to ensure the presence of appropriate laws, regulations, and strategies to address the issue. This recommendation is supported by two subthemes: revising the policies and monitoring and accountability.

#### 3.10.1 Subtheme: Revising the policies

This highlights the need to advocate for updates and revisions to existing policies to address regulatory gaps in medical waste management. For instance, **Respondent 78 (M3PM)** highlighted that “…we realized that our existing policies were outdated and didn’t cover some emerging waste types. We engaged with policymakers to revise and modernize the regulations.”

#### 3.10.2 Subtheme: Monitoring and accountability

This subtheme highlights the establishment of procedures and systems to monitor compliance with waste management rules and policies. As **Interviewee 47 (F3UM)** suggested, “… We introduced a reporting mechanism where any instance of improper disposal was documented. This helped in identifying trends and areas that needed more attention.”

## 4. Discussions

Based on the research findings, the theme that emerged in this section was the challenge of medical waste management in Pakistani districts. This issue was primarily attributed to two interrelated subthemes: poor disposal practices among health professionals and poor waste management among the overall stakeholders. This confirms the findings of the literature that handling medical waste in Pakistan is a persistent concern in Pakistan’s healthcare system and underscores the need for targeted improvement protocols in infrastructure. The knowledge, attitudes, and practices of interns and nursing staff regarding managing biological waste were found to be lacking, according to research carried out at teaching hospitals in District South Karachi [36]. Owing to a lack of knowledge and training, subpar waste management methods may jeopardize the safety of healthcare professionals, patients, and the general public. To guarantee adherence to appropriate waste management practices, thorough training programs must be made available, and healthcare workers must be made more aware of these programs. For instance, in Peshawar, inadequate waste management infrastructure and services hamper the capacity to manage medical waste properly. To guarantee the secure and ethical management of medical waste in Peshawar, the establishment of suitable waste treatment and disposal facilities, including the construction of incinerators or alternative technologies, is required. Medical waste management errors seriously endanger the general public’s health. In Larkana, Sindh Province, Pakistan, there is a severe human immunodeficiency virus (HIV) epidemic, according to a World Health Organization (WHO) study from 2019. The use of contaminated needles, untested blood transfusions, and poor hospital waste management were all blamed for the epidemic [30]. Such occurrences highlight the need to enhance waste management procedures in healthcare institutions to protect the public’s health and avoid outbreaks of the same kind in the future.

The major challenges and effects highlighted were the health and environmental hazards. These were summarized from two sub‑effects: the transmission and infection of diseases to people and soil and water contamination. Community members may be at risk of infection if contaminated waste—such as used needles, bandages, and other biohazardous materials—is not properly disposed of. Infectious illness outbreaks might result from this, putting the native population’s health and welfare at peril. The hazards are further increased by the lack of proper waste segregation and disposal procedures since hazardous waste may mix with routine waste and contaminate water sources and food supplies. This was supported by Sadia et al. [12], who pointed out that the negative impacts of medical waste on the environment may also indirectly affect Indigenous groups, such as limiting their access to clean water and fertile land for agricultural endeavours, which may affect their way of life in the past. The improper treatment of medical waste also impacts the socioeconomic elements of the nearby Indigenous populations.

Another significant consequence was the exacerbation of socioeconomic disparities and inequality, as reflected in the heightened vulnerabilities and financial burdens experienced by certain population groups – particularly children and the elderly. Communities close to improperly managed waste facilities are more likely to be exposed, which adds to the disproportionate burden of health issues. Limited access to healthcare facilities and resources exacerbates health inequities, reducing the effectiveness of treatment and preventative actions for the affected population. According to Baaki et al. [37], the distribution of funds for waste management is another area where socioeconomic disparities are visible. Informal settlements and rural communities are typical examples of places with ineffective waste management systems because they need more financial resources and political clout. Because of this, these underserved areas continue to have gaps in their ability to manage waste, which increases the burden of inappropriate waste disposal on the most vulnerable people. They also need more assistance and investment in waste infrastructure. Health inequalities and socioeconomic inequities brought on by ineffective medical waste management are costs that transcend beyond a particular community or geographic area. For instance, the burden on public health systems and national budgets might include higher healthcare expenses related to treating conditions brought on by exposure to hazardous waste [38].

Indigenous communities were identified as the most affected owing to disproportionate exposure to health risks and vulnerabilities, resulting in a greater impact on their health and well‑being. Their distinct socioeconomic and geographic isolation further amplifies the adverse effects of medical waste on these populations. Indigenous people in the study areas face greater vulnerability to medical waste due to their strong links to the environment, dependence on natural resources, and restricted access to modern waste disposal systems. Improper medical waste handling disproportionately affects Indigenous populations, whose limited access to healthcare prevents timely treatment of infectious diseases caused by hazardous exposures, especially in more remote areas lacking medical facilities near population centers. According to Sadia et al. [12], owing to a lack of access to healthcare services, health issues and, in certain situations, avoidable fatalities may occur more often. Indigenous groups may be more vulnerable to the effects of medical waste owing to their cultural practices and traditions. Medical waste harms humans and animals, pollutes natural resources, threatens traditional livelihoods, and deepens Indigenous disconnection from ancestral lands. Investing in waste management infrastructure in isolated Indigenous areas is crucial. Indigenous communities may considerably reduce the environmental and health concerns associated with hazardous waste disposal by establishing garbage collection and treatment facilities that follow international standards [39].

The study evaluated the measures already undertaken by the government, local organizations, and firms to address the issue of medical waste management. The measures were categorized into two broad themes – regulatory and policy reforms and capacity building and awareness. Regarding the regulatory and policy reforms, the government of Pakistan is implementing necessary policies and regulations that address the issue of poor medical waste management. The legal framework for the appropriate segregation, collection, transportation, and disposal of medical waste in healthcare institutions is provided by the Hospital Waste Management Rules of 2005 [5]. These regulations specify who is accountable for what in the waste management procedures, including healthcare organizations, waste producers, and service suppliers. The government has also developed standard operating procedures (SOPs) and standards for waste management in healthcare institutions. These recommendations outline the correct way to handle hazardous and non‑hazardous components of various forms of medical waste. SOPs specify the procedures for sorting, storing, and transporting garbage, ensuring that healthcare professionals and waste management specialists follow the industry’s best practices. Participants emphasized revising policies and strengthening regulatory enforcement to address challenges in underserved regions effectively. To manage medical waste securely, the government has created waste treatment and disposal facilities in addition to regulatory measures. These facilities handle and eliminate hazardous medical waste using cutting‑edge technology, including autoclaves, microwave systems, and incinerators [6]. It is crucial to expand these technologies to underserved Balochistan to mitigate disease and pollution.

The Pakistani government has also sought public–private partnerships to enhance waste management services. The government wants to improve the country’s garbage collection, transportation, and treatment capacities by working with commercial waste management organizations. The government has also promoted waste management technology research and innovation to find long‑term, low‑cost approaches to managing medical waste. These collaborations may result in better waste management efficiency, fewer negative environmental effects, and better infrastructure. The local government and NGOs have also played a role in ensuring proper medical waste management. These non‑governmental organizations have made various efforts to supplement and assist government activities because they understand how important it is to solve waste management issues. Their efforts have been crucial in advancing sustainable solutions and waste management techniques. Local organizations and NGOs have also built waste‑treatment facilities in locations with inadequate infrastructure [8]. They use cutting‑edge technology to securely manage medical waste, including inexpensive incinerators and biodegradable waste disposal techniques. These programs have successfully lowered the environmental impact of waste disposal and safeguarding the residents’ health.

## 5. Conclusions

The study highlights the adverse impacts of medical waste on Indigenous communities in Balochistan, Pakistan, including health risks, environmental contamination, and socioeconomic disparities. It identifies poor waste management practices and proposes sustainable solutions such as community empowerment, infrastructure improvement, and policy enforcement. The study contributes by emphasizing culturally sensitive, multi‑stakeholder approaches to reduce inequality and enhance resilience in marginalized communities, offering a roadmap for addressing medical waste challenges globally. The recommendations outlined serve as a roadmap towards a more equitable, healthier, and environmentally sustainable future for Indigenous communities in Pakistan. Three main recommendations were developed – (i) empowering Indigenous communities through education and cultural sensitivity, (ii) building strong foundations for infrastructure improvement and collaborative efforts, and (iii) shaping the future through policy revisions, monitoring, and accountability.

The study had some limitations, one of which is its reliance on self‑reported data from interviews and focus group discussions, which may be subject to biases such as social desirability bias or recall bias. Participants may underreport negative practices or overemphasize positive virtues, leading to potential skewing of results and limiting their accuracy. Future studies could include observational methods or triangulation of self‑reported data with objective measures such as waste audits or environmental sampling to enhance the reliability of the results. Another limitation was that the study was geographically limited to three districts in Balochistan, which may not be fully representative of Indigenous communities in Pakistan or other regions with similar issues, especially considering their geographic, cultural, and socioeconomic considerations. Expanding the geographical scope in future studies could provide a better understanding of medical waste management challenges and solutions across diverse Indigenous populations. While the study failed to achieve gender parity with the respondents, the male‑dominated respondents highlighted the greater leadership role played by men in the Indigenous cultures and workplaces, thus making them more knowledgeable to answer the study questions. However, the 35% of respondents who were female may also be considered adequate to present the female perspective on waste management. Notably, the study’s main focus was about documenting the experiences of stakeholders directly involved in waste management practices. Future studies could explore gender‑specific roles in greater detail.
